# Integrated Metabolomic and Transcriptomic Profiles Provide Insights into the Mechanisms of Anthocyanin and Carotenoid Biosynthesis in Petals of *Medicago sativa* ssp. *sativa* and *Medicago sativa* ssp. *falcata*

**DOI:** 10.3390/plants13050700

**Published:** 2024-02-29

**Authors:** Xiuzheng Huang, Lei Liu, Xiaojing Qiang, Yuanfa Meng, Zhiyong Li, Fan Huang

**Affiliations:** Institute of Grassland Research, Chinese Academy of Agricultural Sciences, Hohhot 100081, China; 19917621903@163.com (X.H.); nymyf1990@163.com (Y.M.); zhiyongli1216@126.com (Z.L.); huangfan@caas.cn (F.H.)

**Keywords:** *Medicago sativa* ssp. *sativa*, *Medicago sativa* ssp. *falcata*, transcriptome and metabolome, anthocyanins, carotenoids

## Abstract

The petals of *Medicago sativa* ssp. *sativa* and *M*. *sativa* ssp. *falcata* are purple and yellow, respectively. Free hybridization between *M*. *sativa* ssp. *sativa* and *M*. *sativa* ssp. *falcata* has created hybrids with various flower colors in nature. Moreover, the flower colors of alfalfa are closely correlated with yield, nutritional quality, stress tolerance and other agronomic characteristics. To elucidate the underlying mechanisms of flower color formation in *M*. *sativa* ssp. *sativa* and *M*. *sativa* ssp. *falcata*, we conducted an integrative analysis of the transcriptome and metabolome of alfalfa with three different petal colors (purple, yellow and cream). The metabolic profiles suggested that anthocyanins and carotenoids are the crucial pigments in purple and yellow flowers, respectively. A quantitative exploration of the anthocyanin and carotenoid components indicated that the accumulations of cyanidin, delphinidin, peonidin, malvidin, pelargonidin and petunidin derivatives are significantly higher in purple flowers than in cream flowers. In addition, the content of carotenes (phytoene, α-carotene and β-carotene) and xanthophylls (α-cryptoxanthin, lutein, β-cryptoxanthin, zeaxanthin, antheraxanthin and violaxanthin derivatives) was markedly higher in yellow flowers than in cream flowers. Furthermore, we found that delphinidin-3,5-O-diglucoside and lutein were the predominant pigments accumulated in purple and yellow flowers, respectively. The transcriptomic results revealed that twenty-five upregulated structural genes (one *C4H*, three *4CL*, twelve *CHS*, two *CHI*, one *F3H*, one *F3′H*, one *F3′5′H* and four *DFR*) are involved in the accumulation of anthocyanins in purple flowers, and nine structural genes (two *PSY*, one *ZDS*, two *CRTISO*, two *BCH*, one *ZEP* and one *ECH*) exert an effect on the carotenoid biosynthesis pathway in yellow flowers. The findings of this study reveal the underlying mechanisms of anthocyanin and carotenoid biosynthesis in alfalfa with three classic flower colors.

## 1. Introduction

Alfalfa (*Medicago sativa*) is an important forage, food and ingredient in Chinese herbal medicine, and it is widely planted around the world [1]. It is the most abundantly planted forage, which has excellent traits such as high yield, outstanding nutritional quality, strong hardiness and drought resistance [2,3]. Three alfalfa subspecies show the most typical flower colors, including *M*. *sativa* ssp. *sativa* with purple flowers, *M*. *sativa* ssp. *falcata* with yellow flowers and *M*. *sativa* ssp. *varia* (Martyn) with cream flowers [4]. The flower colors of alfalfa are diverse as *M*. *sativa* ssp. *sativa* and *M*. *sativa* ssp. *falcata* can be freely crossed without reproductive isolation, and the agronomic traits of alfalfa with different flower colors are significantly distinct, such as plant height, leaf area, nutritional quality and stress resistance [5]. Therefore, understanding the potential mechanism underlying the pigment biosynthesis of alfalfa with petals of different colors would be informative with regard to the utilization of and nutritive research on these germplasms.

Flower color, one of the most important phenotypes of flowering plants, provides a visual signal to pollinators and improves pollination efficiency [6]. Plants’ flower color formation is mainly affected by three pigments, including flavonoids, carotenoids and betaines [7]. Anthocyanins are a class of flavonoids and the principal pigments that confer the leaves, petals, peels and seed coats of plants with purple/red/blue/pink colors [8,9]. In addition, anthocyanins can help plants cope with abiotic stress and improve human immunity to prevent diseases [10,11]. Moreover, anthocyanins are frequently combined with one or more glucose, rhamnose, galactose, xylose, arabinose and other glycosides to synthesize various aglycone forms of anthocyanin [12]. The biosynthetic pathway of anthocyanins is generally divided into three stages. This first stage is the phenylpropanoids pathway in which three enzymes, including PAL (phenylalanine ammonia-lyase), C4H (cinnamate-4-hydroxylase) and 4CL (4-coumarate CoA ligase 4), are involved. The second stage is the most crucial stage that affects anthocyanin synthesis, and the core enzymes are CHI (chalcone isomerase), CHS (chalcone synthase), F3H (flavanone 3-hydroxylase), F3′H (flavonoid 3′-hydroxylase) and F3′5′H (flavonoid 3′,5′-hydroxylase). The last stage determines the synthesis and generation of different anthocyanins, which is associated with five enzymes, including DFR (dihydroflavonol 4-reductase), FLS (flavonol synthase), LDOX/ANS (leucoanthocyanidin oxygenase/anthocyanidin synthase), UFGT (UDP-flavonoid glucosyltransferase) and MT (S-adenosyl methionine anthocyaninmethyl transferase) [13,14,15]. In addition, previous studies indicated that the *MYB*, *bHLH*, *WD* and *WRKY* families of transcription factors (TFs) play crucial roles in regulating the anthocyanin anabolic pathway [16,17].

Carotenoids are a class of fat-soluble compounds and the main pigments that produce yellow, orange and red colors [18]. Carotenoids are not only the precursors for abscisic acid (ABA) and strigolactone (SL) biosynthesis, but are also beneficial to human health and nutrition [19,20]. According to their different chemical structures, carotenoids can be divided into two categories, including carotenes and xanthophylls. Carotenes exist in a free state in plants, while xanthophylls combine with different fatty acids in plants to form carotenoids esters [21]. Geranylgeranyl pyrophosphate (GGPP) is a precursor that synthesizes assorted carotenoids via the catalysis of a series of enzymes, including phytoene synthase (PSY), phytoene desaturase (PDS), ζ-carotene desaturase (ZDS), carotenoid isomerase (CRTISO), lycopene ε-cyclase (LCYE), lycopene β-cyclase (LCYB), carotene hydroxylase (BCH), ε-hydroxylase (ECH), violaxanthin de-epoxidase (VDE), zeaxanthin epoxidase (ZEP) and neoxanthin synthase (NXS) [22]. Moreover, the *MYB*, *bHLH*, *AP2/ERF* and *WRKY* families of TFs are essential transcription factors related to flower color that are involved in regulating the carotenoid anabolic pathway [23].

In this study, an analysis of the metabolomes and transcriptomes of the petals of the above-mentioned alfalfa with purple flowers, yellow flowers and cream flowers (hereafter, abbreviated as P, Y and C, respectively) was conducted. Through a comparative analysis of the metabolomes and transcriptomes, we studied the anthocyanin and carotenoid biosynthesis pathways, the anthocyanin and carotenoid contents, and transcription factors that are potentially associated with flower color formation. This work provides insights into the molecular regulatory mechanisms of flower color formation of alfalfa with three different flower colors.

## 2. Results

### 2.1. Targeted Anthocyanin Metabolite Assays of Flowers of Three Different Colors

**Figure 1:**
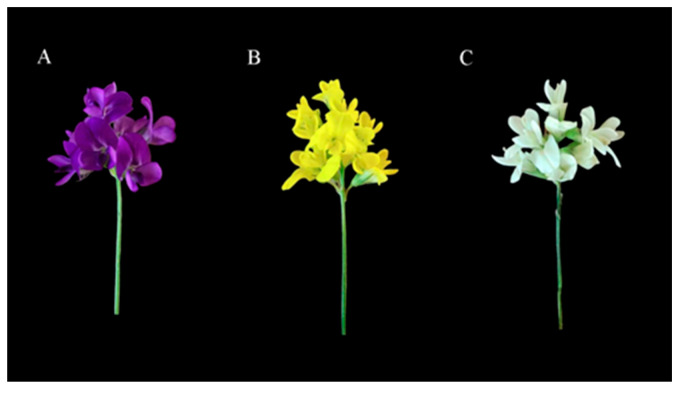
The phenotypes of alfalfa with different flower colors: Alfalfa with (**A**) purple flowers, (**B**) yellow flowers and (**C**) cream flowers.

**Figure 2:**
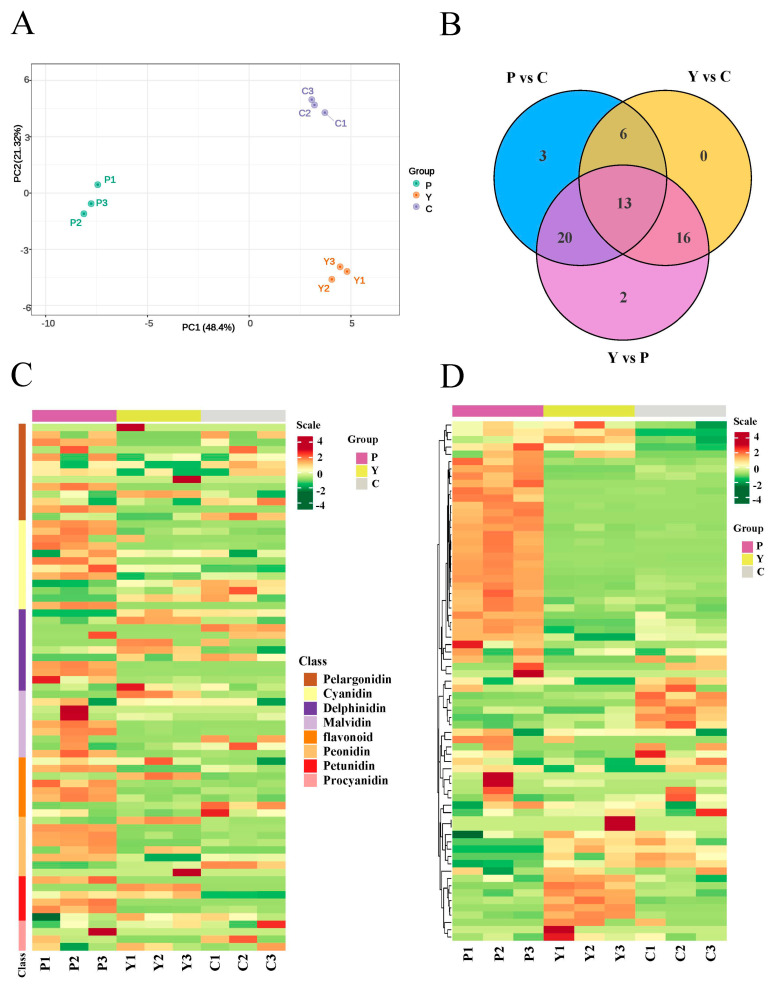
Targeted metabolomic analysis of anthocyanins in the three samples of petals. P1, P2 and P3 represent the three replicates of the P group; Y1, Y2 and Y3 represent the three replicates of the Y group; C1, C2 and C3 represent the three replicates of the C group; Scale: color changes (green to red) represent low to high accumulation. (**A**) PCA plot analysis of anthocyanin metabolome. The *x*-axis represents principal component 1 (PC1); the *y*-axis represents principal component 2 (PC2); (**B**) Venn diagram of DAEs among the three groups. (**C**) Compound class heatmap of anthocyanin DAMs. (**D**) Hierarchical clustering of anthocyanin DAMs.

Previous studies have proven that anthocyanin and carotenoid are essential pigments for the formation of flower color [18,24]. In this work, we established a high-capacity metabolite library using UPLC/MS to explore the anthocyanin and carotenoid content of three types of petals (alfalfa with purple flowers, yellow flowers and cream flowers) (Figure 1). To uncover the accumulation of anthocyanin in these three samples of petals, an analysis of the targeted anthocyanin metabolome of purple flowers, yellow flowers and cream flowers was conducted. Principal component analysis (PCA) showed that the contribution rate of PC1 and PC2 was 48.40% and 21.32%, respectively, and anthocyanin metabolites were clearly separated among the three samples of petals (Figure 2A). These results suggest significant metabolic differences between the three samples of petals.

The compound class heatmap of anthocyanins indicates that a total of 71 differentially accumulated metabolites (DAMs) were identified from the three petal samples and classified into eight categories, including cyanidin (12), delphinidin (11), malvidin (9), pelargonidin (13), peonidin (8), petunidin (6), procyanidin (4) and flavonoid (8) (Figure 2B,C). Moreover, the statistics on the number of differentially accumulated metabolites show that 33 upregulated and 9 downregulated, 15 upregulated and 20 downregulated, and 38 upregulated and 13 downregulated DAMs were identified in the purple flowers vs. cream flowers (P vs. C), the yellow flowers vs. cream flowers (Y vs. C) and the purple flowers vs. yellow flowers (P vs. Y), respectively (Table 1). The hierarchical clustering of anthocyanin DAMs showed that most metabolites in the petals of the purple flowers were significantly up-accumulated in P vs. Y and Y vs. C (Figure 2D). Furthermore, a quantitative analysis of the six anthocyanins (cyanidin, delphinidin, malvidin, pelargonidin, peonidin and petunidin) content displayed that six metabolites maintained significantly high content in the P group (c > 30 μg·g^−1^), including cyanidin-3,5-O-diglucoside (104.71 μg·g^−1^), delphinidin-3,5-O-diglucoside (5186.38 μg·g^−1^), delphinidin-3-O-glucoside (148.64 μg·g^−1^), malvidin-3,5-O-diglucoside (117.03 μg·g^−1^), pelargonidin-3,5-O-diglucoside (50.93 μg·g^−1^) and petunidin-3-O-glucoside (32.23 μg·g^−1^), while the content of these metabolites was low in the Y and C, ranging from 0 to 27.22 μg·g^−1^ (Table S1). In addition to the six anthocyanins, the content of other anthocyanins was low in the Y and C groups, ranging from 0 to 2.18 μg·g^−1^ (Table S1). These results reveal that anthocyanins might play crucial roles in the color formation of purple flowers.

### 2.2. Anthocyanin Components Accumulated in Purple Flowers and Cream Flowers

**Figure 3:**
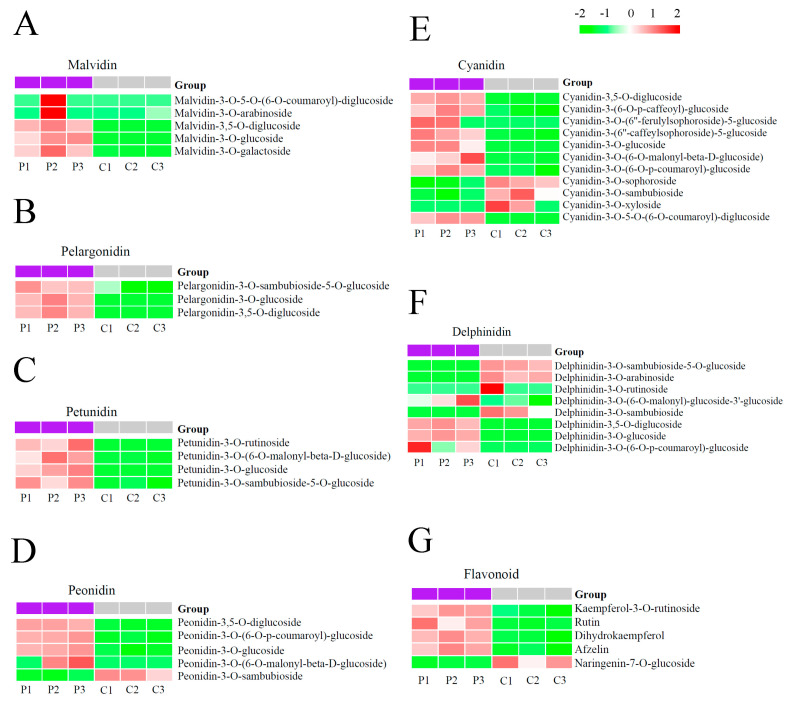
The DAE heatmap of malvidin (**A**), pelargonidin (**B**), petunidin (**C**), peonidin (**D**), cyanidin (**E**), delphinidin (**F**) and flavonoid (**G**) in purple flowers vs. cream flowers. P1, P2 and P3 represent the three replicates of the P group; C1, C2 and C3 represent the three replicates of the C group; low to high accumulation is suggested by color changes (green to red).

To elucidate which anthocyanins might play a major role in the color formation of purple flowers, we investigated the results of the quantitative analysis and the DAE heatmap between the purple flowers and cream flowers. The DAE heatmap showed that the accumulations of malvidins (malvidin-3-O-5-O-(6-O-coumaroyl)-diglucoside, malvidin-3-O-arabinoside, malvidin-3,5-O-diglucoside, malvidin-3-O-glucoside and malvidin-3-O-galactoside) (Figure 3A), pelargonidins (pelargonidin-3-O-sambubioside-5-O-glucoside, pelargonidin-3-O-glucoside and pelargonidin-3,5-O-diglucoside) (Figure 3B), petunidins (petunidin-3-O-rutinoside, petunidin-3-O-(6-O-malonyl-beta-D-glucoside, petunidin-3-O-glucoside and petunidin-3-O-sambubioside-5-O-glucoside) (Figure 3C), peonidins (peonidin-3,5-O-diglucoside, peonidin-3-O-(6-O-*p*-coumaroyl)-glucoside, peonidin-3-O-glucoside, and peonidin-3-O-(6-O-malonyl-beta-D-glucoside)) (Figure 3D), cyanidins (cyanidin-3,5-O-diglucoside, cyanidin-3-(6-O-*p*-caffeoyl)-glucoside, cyanidin-3-O-(6″-ferulylsophoroside)-5-glucoside, cyanidin-3-(6″-caffeylsophoroside)-5-glucoside, cyanidin-3-O-glucoside, cyanidin-3-O-(6-O-malonyl-beta-D-glucoside), cyanidin-3-O-(6-O-*p*-coumaroyl)-glucoside, cyanidin-3-O-5-O-(6-O-coumaroyl)-diglucoside) (Figure 3E) and delphinidins (delphinidin-3-O-(6-O-malonyl)-glucoside-3′-glucoside, delphinidin-3,5-O-diglucoside, delphinidin-3-O-glucoside, delphinidin-3-O-(6-O-*p*-coumaroyl)-glucoside) (Figure 3F) exhibited higher content level in the P group compared to the C group. These results illustrate that these malvidin, pelargonidin, petunidin, peonidin, cyanidin and delphinidin derivatives might participate in the color formation of purple flowers.

Among these anthocyanin derivatives, a quantitative analysis showed that some metabolites maintain a high level in the P group and are significantly up-accumulated in P vs. C (Table S2), such as delphinidin-3,5-O-diglucoside (c = 5186.38 μg·g^−1^; Log_2_fold change values = 8.43), delphinidin-3-O-glucoside (c = 148.64 μg·g^−1^; Log_2_fold change values = 7.49), malvidin-3,5-O-diglucoside (c = 117.03 μg·g^−1^; Log_2_fold change values = 6.36), cyanidin-3,5-O-diglucoside (c = 104.71 μg·g^−1^; Log_2_fold change values = 4.59), pelargonidin-3,5-O-diglucoside (c = 50.93 μg·g^−1^; Log_2_fold change values = 7.29), petunidin-3-O-glucoside (c = 32.23 μg·g^−1^; Log_2_fold change values = inf), cyanidin-3-(6-O-*p*-caffeoyl)-glucoside (c = 4.69 μg·g^−1^; Log_2_fold change values = 1.59) and peonidin-3,5-O-diglucoside (c = 3.91 μg·g^−1^; Log_2_fold change values = 2.99) (Table S2). These results indicate that these metabolites might be the key anthocyanins involved in the color formation of purple flowers. Interestingly, we discovered that the content of delphinidin-3,5-O-diglucoside accounted for more than 91.63% of the total anthocyanins in purple flowers (Figure S1), suggesting that delphinidin-3,5-O-diglucoside plays a more essential role in the color formation of purple flowers than other anthocyanins.

Additionally, we identified that four flavonoids (kaempferol-3-O-rutinoside, Log_2_fold change values = 1.22; rutin, Log_2_fold change values = 2.00; dihydrokaempferol Log_2_fold change values = 1.97; afzelin, Log_2_fold change values = 1.78) and one flavonoid (naringenin-7-O-glucoside, Log_2_fold change values = −1.87) were notably up-accumulated and down-accumulated in the purple flowers as compared to the cream flowers (Figure 3G, Table S2), respectively, suggesting that these flavonoids might be closely associated with anthocyanin accumulation in purple flowers.

### 2.3. Targeted Carotenoid Metabolite Assays of Flowers of Three Different Colors

**Figure 4:**
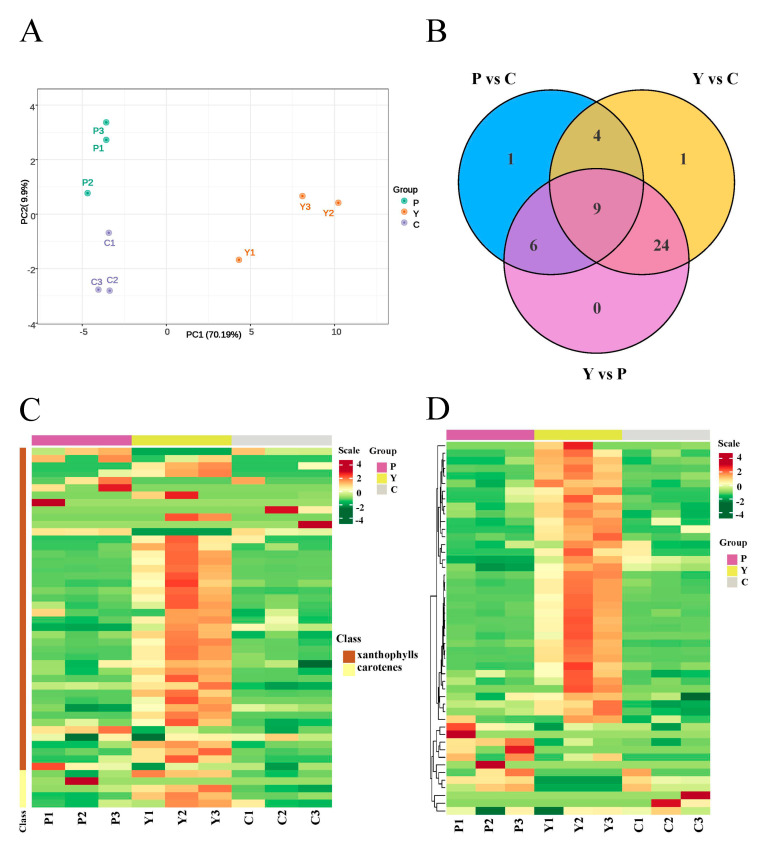
Targeted metabolomic analysis of carotenoids in the three samples of petals. P1, P2 and P3 represent the three replicates of the P group; Y1, Y2 and Y3 represent the three replicates of the Y group; C1, C2 and C3 represent the three replicates of the C group; Scale: color changes (green to red) represent low to high accumulation. (**A**) PCA plot analysis of carotenoid metabolome. The *x*-axis represents principal component 1 (PC1); the *y*-axis represents principal component 2 (PC2); the three samples (P, Y and C) are distinguished by different colors. (**B**) Venn diagram of DAEs among the three groups. (**C**) Compound class heatmap of carotenoid DAMs. (**D**) Hierarchical clustering of carotenoid DAMs.

To further explore whether carotenoids contribute to the color formation of the three alfalfa germplasms with different flower colors, targeted carotenoid metabolomics was performed on their petals. As shown in Figure 4A, the PCA chart displays evident separation of the samples based on flower color, suggesting that the experiment was reliable and reproducible.

The compound class heatmap of carotenoids indicates that a total of 49 differentially accumulated metabolites (DAMs) were identified from the three petal samples (Figure 4B) and classified into two categories: carotenes (5) and xanthophylls (44) (Figure 4C). Among these DAMs, we detected 11 upregulated and 9 downregulated DAMs, 33 upregulated and 5 downregulated DAMs, and 32 upregulated and 7 downregulated DAMs in the P vs. C comparison, the Y vs. C comparison and the P vs. Y comparison (Table 2), and the hierarchical clustering of carotenoid DAMs clearly showed that most metabolites maintain a higher content in the petals of the yellow flowers compared to that in the purple and cream flowers (Figure 4D).

Moreover, a quantitative analysis of the carotenoids content indicated that the accumulation of some metabolites maintains significantly high levels in the Y group, such as lutein myristate (9.01 μg·g^−1^), lutein dimyristate (8.35 μg·g^−1^), lutein (60.76 μg·g^−1^), lutein dilaurate (5.15 μg·g^−1^), violaxanthin palmitate (4.00 μg·g^−1^) and zeaxanthin dipalmitate (7.89 μg·g^−1^), while the content of these metabolites was low in the P and C groups (Table S3). In addition to the six carotenoids, the content of remaining carotenoids was also very low in the P and C groups, ranging from 0 to 2.41 μg·g^−1^ (Table S3). Additionally, the content of five down-accumulated carotenoids (β-cryptoxanthin myristate, canthaxanthin, rubixanthin caprate, antheraxanthin dipalmitate and β-cryptoxanthin laurate) in Y vs. C was very low in the C group, ranging from 0 to 0.059 μg·g^−1^ (Table S3). Simultaneously, the accumulation of seven down-accumulated carotenoids (β-cryptoxanthin oleate, γ-carotene, β-cryptoxanthin myristate, canthaxanthin, 5,6epoxy-lutein-caprate-palmitate, lutein distearate and β-cryptoxanthin laurate) in Y vs. P was very low in the P group, ranging from 0.00023 to 0.060 μg·g^−1^ (Table S3). These results revealed that carotenoids likely facilitate the yellow pigmentation of petals.

### 2.4. Identification of Carotenoid Components Involved in the Color Formation of Yellow Flowers

**Figure 5:**
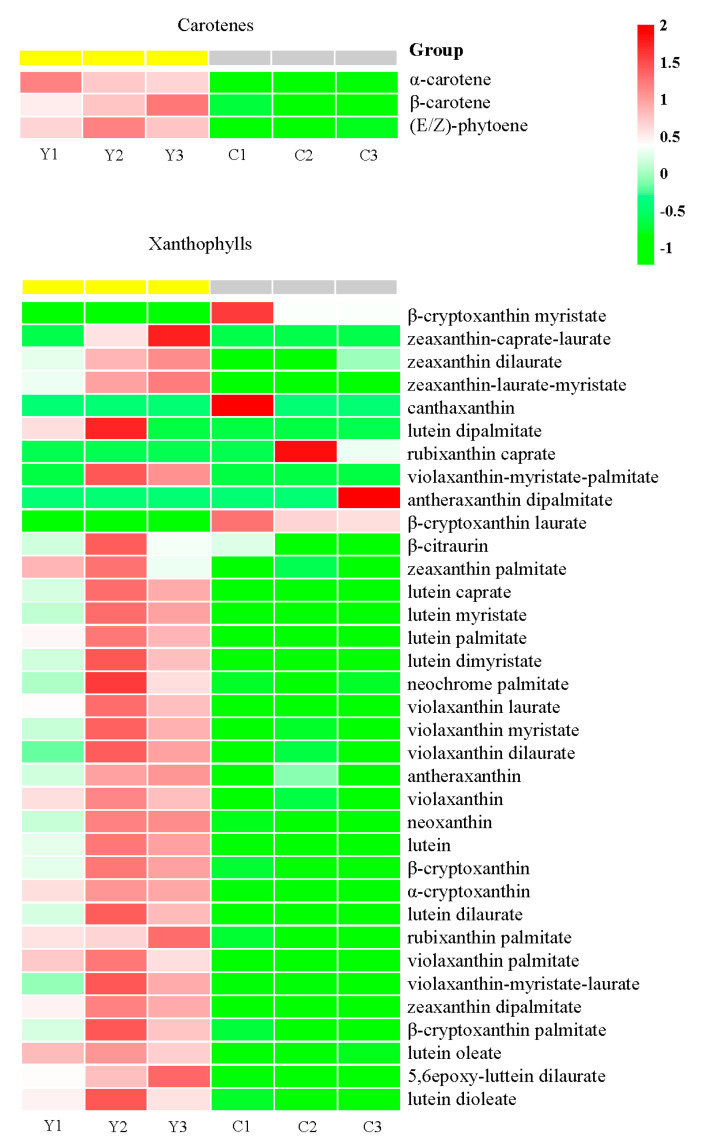
DAE heatmap of carotenes and xanthophylls in yellow flowers vs. cream flowers. Y1, Y2 and Y3 represent the three replicates of the Y group; C1, C2 and C3 represent the three replicates of the C group; low to high accumulation is suggested by color changes (green to red).

Subsequently, we investigated the results of the quantitative analysis and the DAE heatmap of the yellow flowers and cream flowers to explore which carotenoids might give rise to the color formation of the yellow flowers. Then, we detected that the accumulation of three carotenes (α-carotene, Log_2_fold change values = 1.13; β-carotene, Log_2_fold change values = 1.15; (E/Z)-phytoene, Log_2_fold change values = 1.77) was significantly higher in the Y group than in the C group (Figure 5, Table S4).

Additionally, we found that ten lutein derivatives were significantly up-accumulated in Y vs. C, including lutein (Log_2_fold change values = 2.04), lutein myristate (Log_2_fold change values = 4.27), lutein dimyristate (Log_2_fold change values = 5.14), lutein palmitate (Log_2_fold change values = 3.61), lutein dilaurate (Log_2_fold change values = 4.47), lutein dipalmitate (Log_2_fold change values = 5.57), lutein caprate (Log_2_fold change values = 3.60), lutein oleate (Log_2_fold change values = 3.19), 5,6epoxy-luttein dilaurate (Log_2_fold change values = 4.40) and lutein dioleate (Log_2_fold change values = 4.86) (Figure 5, Table S4). 

Moreover, five zeaxanthin (zeaxanthin-caprate-laurate, zeaxanthin dilaurate, zeaxanthin-laurate-myristate, zeaxanthin palmitate and zeaxanthin dipalmitate) and seven violaxanthin derivatives (violaxanthin, violaxanthin-myristate-palmitate, violaxanthin laurate, violaxanthin myristate, violaxanthin dilaurate, violaxanthin palmitate and violaxanthin-myristate-laurate) were up-accumulated in Y vs. C, ranging from 1.25-fold to 4.77-fold increments (Figure 5, Table S4).

In addition, the content of β-cryptoxanthin (Log_2_fold change values = 1.68), β-cryptoxanthin palmitate (Log_2_fold change values = 2.06), α-cryptoxanthin (Log_2_fold change values = 4.86), antheraxanthin (Log_2_fold change values = 1.80) and neoxanthin (Log_2_fold change values = 1.12) was significantly higher in the yellow flowers compared to the cream flowers (Figure 5, Table S4).

Furthermore, we found that some DAEs maintained a high content in the yellow flowers, such as lutein (60.76 μg·g^−1^), zeaxanthin (12.06 μg·g^−1^), lutein myristate (9.01 μg·g^−1^), lutein dimyristate (8.35 μg·g^−1^), zeaxanthin dipalmitate (7.89 μg·g^−1^) and violaxanthin-myristate-laurate (6.31 μg·g^−1^) (Table S4). The results indicate that these metabolites might be the key carotenoids participating in the color formation of the yellow flowers. Notably, lutein accounted for more than 39.23% of the total carotenoids enriched in the yellow flowers, suggesting that lutein may be the most essential carotenoids contributing to the color formation of yellow flowers (Figure S2).

### 2.5. Transcriptome Analysis of Flower Petals of the Three Different Colors

To uncover the molecular mechanism of color formation in the three samples of petals, an analysis of the transcriptome of purple flowers, yellow flowers and cream flowers was performed. Then, we conducted a comparative analysis of the purple flowers vs. yellow flowers, the purple flowers vs. cream flowers and the yellow flowers vs. cream flowers. After cleaning and quality checking, the RNA-seq obtained 59.21 Gb of clean reads, with no less than 5 Gb of clean reads per library. The average percentage of the Q30 bases was 94%. More than 73% of the clean reads from the nine biological replicates of the three petal samples could be uniquely mapped to the reference genome (Table S5). These transcriptome results suggested that the RNA-sequencing datasets were reliable for further research. According to the FPKM values, the hierarchical clustering of DEGs revealed that the gene expression patterns were different among the three alfalfa species (Figure S3). The volcano maps showed that 4816 upregulated and 5045 downregulated genes, 5686 upregulated and 5908 downregulated genes, and 4816 upregulated and 5045 downregulated genes were detected in the P vs. Y comparison, P vs. C comparison and Y vs. C comparison, respectively (Figure S4). The KEGG analysis indicated that many DEGs were enriched in anthocyanin accumulation-related pathways in the P vs. C comparison, such as phenylpropanoid biosynthesis (ko00940), flavonoid biosynthesis (ko00941), isoflavonoid biosynthesis (ko00943), and flavone and flavonol biosynthesis (ko00944), and some DEGs were significantly enriched in carotenoid biosynthesis (ko00906) in the Y vs. C comparison (Figure S5).

To confirm the reliability of the RNA-Seq results, we selected ten crucial genes participating in the anthocyanin and carotenoid biosynthetic pathways to conduct qRT-PCR. The relative expression levels of the genes were similar to the RNA-seq results, which suggested that the RNA-Seq data were reliable in this work (Figure S6).

### 2.6. Expression Analysis of Anthocyanin Metabolic Pathway Genes

**Figure 6:**
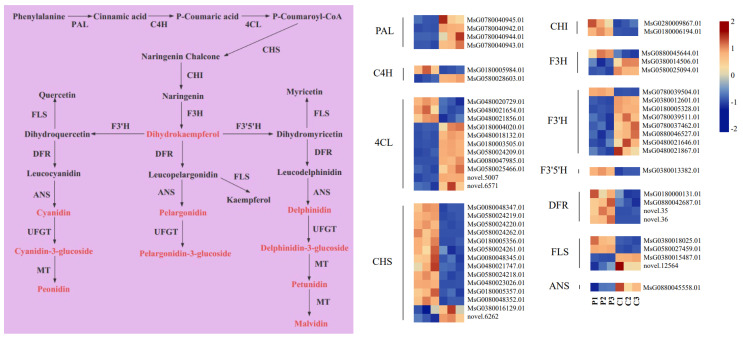
Structural genes that participate in the anthocyanin pathway of purple flowers and cream flowers. A low to high expression is suggested by color changes (blue to red). PAL, phenylalanine ammonia-lyase; C4H, cinnamate-4-hydroxylase; 4CL, 4-coumarate CoA ligase 4; CHS, chalcone synthase; CHI, chalcone isomerase; F3H, flavanone 3-hydroxylase; F′H, flavonoid 3′-hydroxylase; F3′5′H, flavonoid 3′,5′-hydroxylase; DFR, dihydroflavonol 4-reductase; UFGT, UDP-flavonoid glucosyltransferase; ANS, anthocyanidin synthase; MT, S-adenosyl methionine anthocyanin methyl transferase.

By examining the transcriptomic results, an anthocyanins biosynthesis pathway was constructed to further understand the underlying mechanism of anthocyanin accumulation in the purple flowers (Figure 6). A total of 55 structural genes related to anthocyanin biosynthesis were selected, including *PAL* (4), *C4H* (2), *4CL* (11), *CHS* (14), *CHI* (2), *F3H* (3), *F3′H* (8), *F3′5′H* (1), *DFR* (4), *FLS* (4), *ANS* (1) and *ANR* (1); these genes were significantly differentially expressed in the purple flowers vs. the cream flowers.

Among these DEGs, one *C4H* (*MsG0180005984.01*, Log_2_fold change values = 3.67), three *4CL* (*MsG0480020729.01*, Log_2_fold change values = 2.61; *MsG0480021654.01*, Log_2_fold change values = 1.63; *MsG0480021856.01*, Log_2_fold change values = 1.42), twelve *CHS* (*MsG0080048347.01*, Log_2_fold change values = 3.63; *MsG0580024219.01*, Log_2_fold change values = 2.06; *MsG0580024220.01*, Log_2_fold change values = 3.07; *MsG0580024262.01*, Log_2_fold change values = 6.47; *MsG0180005356.01*, Log_2_fold change values = 3.56; *MsG0580024261.01*, Log_2_fold change values = 2.10; *MsG0080048345.01*, Log_2_fold change values = 2.38; *MsG0480021747.01*, Log_2_fold change values = 3.44; *MsG0580024218.01*, Log_2_fold change values = 2.37; *MsG0480023026.01*, Log_2_fold change values = 1.37; *MsG0180005357.01* Log_2_fold change values = 1.15; *MsG0080048352.01*, Log_2_fold change values = 4.02), two *CHI* (*MsG0280009867.01*, Log_2_fold change values = 2.29; *MsG0180006194.01*, Log_2_fold change values = 1.23), one *F3H* (*MsG0880045644.01*, Log_2_fold change values = 1.59), one *F3′H* (*MsG0780039504.01*, Log_2_fold change values = 2.20), one *F3′5′H* (*MsG0380013382.01,* Log_2_fold change values = 1.79) and four *DFR* (*MsG0180000131.01*, Log_2_fold change values = 1.60; *MsG0880042687.01*, Log_2_fold change values = 1.82; *novel.35*, Log_2_fold change values = 5.65; *novel.36*, Log_2_fold change values = 7.79) genes were significantly upregulated in purple flowers in comparison to cream flowers, and these DEGs were positively associated with anthocyanin accumulations in the purple flowers.

### 2.7. Expression Analysis of Carotenoid Metabolic Pathway Genes

**Figure 7:**
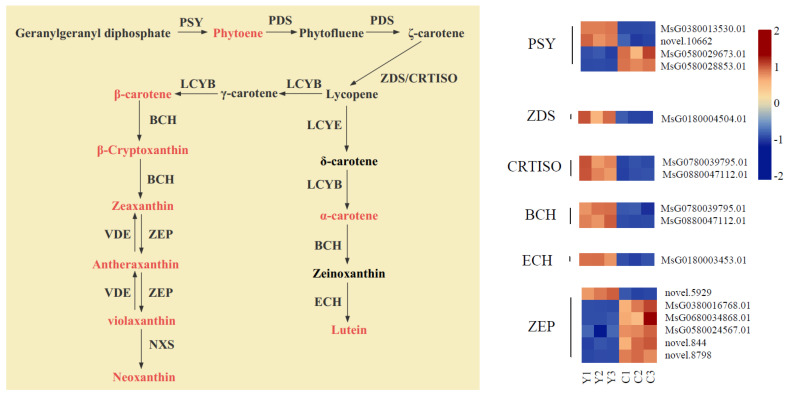
Structural genes that participate in the carotenoid biosynthesis pathway of yellow flowers and cream flowers. A low to high expression is suggested by color changes (blue to red). PSY, phytoene synthase; PDS, phytoene desaturase; ZDS, ζ-carotene desaturase; CRTISO, carotenoid isomerase; LCYE, lycopene ε-cyclase; LCYB, lycopene β-cyclase; BCH, carotene hydroxylase, ECH, ε-hydroxylase; ZEP, zeaxanthin epoxidase; VDE, violaxanthin de-epoxidase; NXS, neoxanthin synthase.

Simultaneously, we established a carotenoid biosynthesis pathway to explore the molecular mechanism of carotenoid accumulation in the yellow flowers (Figure 7). By analyzing the differences in gene expression levels between the yellow flowers and cream flowers, we detected a total of 16 DEGs related to carotenoid biosynthesis, including *PSY* (4), *ZDS* (1), *CRTISO* (2), *BCH* (2), *ZEP* (6) and *ECH* (1).

Among these DEGs, two *PSY* (*MsG0380013530.01*, Log_2_fold change values = 7.91; *novel.10662*, Log_2_fold change values = 3.30), one *ZDS* (*MsG0180004504.01*, Log_2_fold change values = 1.50), two *CRTISO* (*MsG0880045723.01*, Log_2_fold change values = 2.32; *MsG0180003155.01*, Log_2_fold change values = 1.73), two *BCH* (*MsG0780039795.01*, Log_2_fold change values = 1.25; *MsG0880047112.01*, Log_2_fold change values = 1.53), one *ZEP* (*novel.5929*, Log_2_fold change values = 3.70) and one *ECH* (*MsG0180003453.01*, Log_2_fold change values = 2.08) genes were notably upregulated in the yellow flowers compared to the cream flowers. Thus, these structural genes might be involved in carotenoid accumulation in yellow flowers.

### 2.8. Identification of Transcription Factors Related to Anthocyanin and Carotenoid Biosynthesis

Transcription factors (TFs), also known as trans-acting factors, regulate plant development, abiotic stress response and the biosynthesis of secondary metabolites by changing gene expression [25,26,27]. In our studies, a total of 584 differentially expressed TFs were identified in the purple flowers vs. cream flowers. Anthocyanin biosynthesis was primarily regulated by *MYB*, *bHLH* and *WRKY* TFs [17]. Among these, we identified *MYB* (49), *bHLH* (24) and *WRKY* (27) differentially expressed TFs, which might play an essential role in the regulation of candidate genes related to anthocyanin biosynthesis in purple flowers. 

A previous study suggested that *MYB*, *bHLH*, *AP2/ERF* and *WRKY* TFs are the essential transcription factors related to flower color involved in regulating the carotenoid anabolic pathway [23]. By comparing the yellow flowers and the cream flowers, we identified a total of 582 differentially expressed TFs, including *MYB* (52), *bHLH* (24), *AP2/ERF* (44), *WRKY* (23) and other TFs. These TFs may participate in carotenoid biosynthesis in yellow flowers.

## 3. Discussion

The colors of alfalfa flowers are spectacularly diverse in nature, such as purple, yellow, red, blue, green, pink, gray and cream [5,28]. Alfalfa varieties with three representative flower colors—purple, yellow and cream—were selected to study the mechanisms of flower color formation in this work. By analyzing metabolomic data, we discovered that anthocyanins and carotenoids are involved in the color formation of purple and yellow flowers, respectively. Using transcriptomic analysis, we uncovered some DEGs associated with anthocyanin biosynthesis and carotenoid biosynthesis that probably participate in pigment accumulation. Furthermore, an integrative transcriptome and metabolome analysis was performed to reveal the mechanism of alfalfa flower coloration.

### 3.1. Component and Content of Anthocyanins and Carotenoids in Petals of the Three Different Colors

Anthocyanins are the crucial pigments that endow petals with various colors, and commonly include cyanidin, delphinidin, malvidin, pelargonidin, peonidin and petunidin [29]. A previous study suggested that malvidin and petunidin derivatives are the major anthocyanins found in the purple flowers of alfalfa [30]. In our present work, we observed that in addition to malvidin and petunidin derivatives, cyanidin, pelargonidin, peonidin and delphinidin derivatives are also the essential anthocyanins in the petals of purple flowers.

In recent years, many studies have reported that carotenoids are closely correlated with yellow color formation in plant tissues [31,32,33]. In agreement with previous findings, we uncovered that most carotenoids are highly accumulated in yellow flowers compared to cream flowers, including α-carotene, β-carotene, (E/Z)-phytoene, α-cryptoxanthin, lutein and its derivatives, β-cryptoxanthin and its derivatives, zeaxanthin derivatives, violaxanthin and its derivatives, antheraxanthin and neoxanthin. The results reveal that these carotenoids are involved in the color formation of yellow flowers.

### 3.2. Key Genes That Participate in Anthocyanin Biosynthesis in Purple Flowers

Key genes participating in anthocyanin biosynthesis in petals have been studied [34,35]. Flavonoids are synthesized by using phenylalanine as a precursor, and PAL, C4H and 4CL can convert phenylalanine to 4-coumaroyl-CoA. In this work, we found that the genes, *C4H* (*MsG0180005984.01*) and *4CL* (*MsG0480020729.01*, *MsG0480021654.01* and *MsG0480021856.01*), are highly expressed in purple flowers in comparison to cream flowers. These four genes are associated with the synthesis of 4-coumaroyl-CoA. Subsequently, 4-coumaroyl-CoA is catalyzed by three enzymes (CHS, CHI and F3H) to synthesize dihydrokaempferol. The CHS enzyme is the first committed step in the biosynthesis of anthocyanins, which involves the conversion of 4-coumaroyl-CoA to naringenin chalcone. Deng et al. used virus-induced gene silencing (VIGS) to knock out *GCHS1* in gerbera and obtained transgenic strains with lighter flowers [36]. In the present study, we identified twelve genes of *CHS* that were highly expressed in purple flowers more than in cream flowers; therefore, the low expression levels of *CHS* might be the primary reason for the loss of six anthocyanins in cream flowers. Afterwards, 4-coumaroyl-CoA is converted to naringenin by CHI. In this work, we found that two *CHI genes* (*MsG0280009867.01* and *MsG0180006194.01*) were significantly upregulated in purple flowers, suggesting that *CHI* might be involved in anthocyanin biosynthesis in purple flowers. Moreover, the high expression of *F3H* supplied an adequate amount of dihydrokaempferol. The metabolomic analysis showed that dihydrokaempferol accumulation in purple flowers (32.63 μg·g^−1^) is about four times higher than in cream flowers (8.31 μg·g^−1^). Concurrently, we detected that the gene, *F3H* (*MsG0880045644.01*, Log_2_ fold-change value = 1.59), was significantly upregulated in purple flowers compared to cream flowers. These results indicate that the gene, *F3H* (*MsG0880045644.01*), plays a critical role in dihydrokaempferol accumulation in purple flowers. 

In the next step, F3′H catalyzes dihydrokaempferol to dihydroquercetin, which is a precursor in the synthesis of cyanidin and peonidin. In the present study, we detected that the gene, *F3′H* (*MsG0780039504.01*, Log_2_ fold-change value = 2.20), is highly expressed in purple flowers compared to cream flowers. The results indicate that F3′H may participate in the synthesis of cyanidin and peonidin derivatives. Dihydrokaempferol is also catalyzed to dihydromyricetin by F3′5′H. Dihydromyricetin is a precursor in the synthesis of delphinidin, petunidin and malvidin. Previous research has suggested that delphinidin, petunidin and malvidin can endow plant tissues and organs with a purple color, and the gene, *F3′5′H*, is frequently associated with purple color formation [37,38]. In the present work, we detected that the gene, *F3′5′H* (*MsG0380013382.01*, Log_2_ fold-change value = 1.80), is significantly upregulated in purple flowers compared to cream flowers. At the same time, the content of delphinidin, petunidin and malvidin derivatives was found to be significantly higher in purple flowers than in cream flowers. These results reveal that the gene, *F3′5′H*, plays an essential role in the accumulations of delphinidin, petunidin and malvidin. Additionally, the metabolic analysis indicated that delphinidin-3,5-O-diglucoside is the most essential anthocyanin involved in the color formation of purple flowers. Based on the above results, it is reasonable to speculate that *F3*′*5*′*H* is the most critical gene associated with the color formation of purple flowers. 

DFR is a key enzyme and can catalyze dihydrokaempferol, dihydroquercetin and dihydromyricetin to leucopelargonidin, leucocyanidin and leucodelphinidin, respectively. Most studies have demonstrated that *DFR* is the critical gene correlated with anthocyanin biosynthesis in plant tissues and organs [35,39]. In this work, we discovered four *DFR* genes that were highly expressed in purple flowers compared to cream flowers. These results indicate that the highly expressed levels of *DFR* are closely associated with the accumulations of various anthocyanins in purple flowers. The high expression levels of the gene, *FLS*, increased flavone and flavonol accumulations and negatively regulated anthocyanin biosynthesis [40,41]. In this study, the high expression of two *FLS* genes was identified in cream flowers compared to purple flowers; however, the quantitative analysis of metabolites showed that flavone and flavonol (kaempferol-3-O-rutinoside, rutin and afzelin) are significantly up-accumulated in purple flowers compared to cream flowers. Therefore, we concluded that FLS does not contribute significantly to anthocyanin biosynthesis in cream flowers. 

### 3.3. Key Genes That Participate in Carotenoid Biosynthesis in Yellow Flowers

PSY is the first enzyme involved in carotenoid biosynthesis, and it catalyzes geranylgeranyl pyrophosphate to phytoene [42]. *Eschscholzia californica* is known for its bright golden-orange flowers, and the deletion of *PSY* prevents the synthesis of carotenoids in white cultivars [43]. In this study, two *PSY* genes (*MsG0380013530.01*, Log_2_ fold-change value = 7.91; *novel.10662*, Log_2_ fold-change value = 3.30) were significantly upregulated in yellow flowers compared to cream flowers, and phytoene maintained a high content in yellow flowers compared to cream flowers. These results indicate that PSY plays a critical role in phytoene synthesis in yellow flowers. After phytoene is catalyzed to ζ-carotene by PDS, ZDS and CRTISO can catalyze ζ-carotene to lycopene. Li [44] confirmed that high expression levels of the genes, *CRTISO* and *ZDS*, can increase carotenoid accumulation for golden-leaf coloration in the mutant *G. biloba*. In this study, we detected that one *ZDS* gene (*MsG0180004504.01*, Log_2_ fold-change value = 1.50) and two *CRTISO* genes (*MsG0880045723.01*, Log_2_ fold-change value = 2.32; *MsG0180003155.01*, Log_2_ fold-change value = 1.73) are significantly upregulated in yellow flowers compared to cream flowers. The results suggest that higher expression levels of the *ZDS* and *CRTISO* genes may facilitate carotenoid accumulations in yellow flowers. During carotenoid biosynthesis, BCH, an important enzyme involved in carotenoid biosynthesis, can catalyze α-carotene, β-carotene and β-cryptoxanthin to zeinoxanthin, β-cryptoxanthin and zeaxanthin, respectively. Moreover, a previous study revealed that a high expression level of *BCH* is associated with the yellow color formation of petals [45]. In the present work, we observed that two *BCH* genes (*MsG0780039795.01*, Log2 fold-change value = 1.25; *MsG0880047112.01*, Log2 fold-change value = 1.53) are significantly upregulated in yellow flowers compared to cream flowers. Combined with the results of the metabolic measurements, we found that zeinoxanthin, β-cryptoxanthin and zeaxanthin maintain high levels in yellow flowers in comparison to cream flowers. These results suggest that *BCH* genes positively participate in the accumulations of β-cryptoxanthin and zeaxanthin. Subsequently, zeinoxanthin is catalyzed to lutein by ECH. Our metabolic analysis showed that lutein is significantly up-accumulated in yellow flowers compared to cream flowers, and the expression of one *ECH* gene (*MsG0180003453.01*, Log_2_ fold-change value = 2.08) showed a higher expression level in yellow flowers than in cream flowers. Therefore, we concluded that higher expression levels of the *ECH* gene may contribute to lutein accumulations and eventually lead to the color formation of yellow flowers.

### 3.4. TFs Related to Anthocyanin and Carotenoid Biosynthesis

TFs of the *MYB* family play a vital role in the regulation of anthocyanin biosynthesis [46]. For example, Zhou et al. observed that *CmMYB3*-like negatively regulated anthocyanin biosynthesis in *Chrysanthemum morifolium* [47]. *bHLH* TFs are associated with *MYB* and *WD* to regulate anthocyanins’ biosynthesis [48]. In addition, *WRKY* TFs responsible for regulating anthocyanin biosynthesis have been reported [49]. Among all the differentially expressed genes, we identified 49 *MYB*, 24 *bHLH* and 27 *WRKY* TFs in the comparison of purple flowers vs. cream flowers. These results will enable the clarification of the functions of TFs during anthocyanin biosynthesis in purple flowers.

The transcription factor, *AP2.2*, interacts with its partner *SINAT2* to negatively regulate the expression level of the gene, *PSY*, and decrease the carotenoid content in *Arabidopsis* leaves [50]. Moreover, previous studies suggested TFs of the *R2R3-MYB, bHLH* and *WRKY* families are also involved in regulating carotenoid biosynthesis [51,52,53]. In the current study, 52 *MYB*, 24 *bHLH*, 44 *AP2/ERF*, 23 *WRKY* TFs with differential expression were identified in the comparison of yellow flowers vs. cream flowers. These TFs may have essential functions in controlling carotenoid biosynthesis in yellow flowers.

## 4. Materials and Methods

### 4.1. Plant Materials

The three samples (alfalfa with purple flowers, yellow flowers and cream flowers) were cultivated at the Institute of grassland research of the Chinese Academy of Agricultural Sciences in Hohhot (40°58′ N, 111°78′ E). All samples were planted in the same environment and under the same management practices. We collected petals of each color from the same stem when the plants reached the blooming stage. We named the petals of the purple flowers ‘P’, those of the yellow flowers ‘Y’ and those of the cream flowers ‘C’. Three biological replicates were obtained for the three samples, and the petals of each color were taken from 10 flowers and pooled for each biological replicate.

### 4.2. Transcriptome Sequencing and Data Analysis

The total RNA of the petals (P, Y and C) was extracted using ethanol precipitation and CTAB-PBIOZO. The quality and preliminary quantification of mRNAs were detected using an Agilent 2100 Bioanalyzer (Agilent Technologies, Baden-Württemberg, Germany) and a Qubit 2.0 Fluorometer (Thermo Fisher Scientific, Waltham, MA, USA), respectively. Nine cDNA libraries were sequenced by utilizing the Illumina sequencing 6000 platform. Clean reads were acquired after low-quality sequences were withdrawn and then assembled using the Fastp v0.19.3. All non-redundant transcripts were mapped to the *M. sativa* reference genome (https://figshare.com/articles/dataset/M_sativa_genome_and_annotation_files/12623960 (accessed on 20 October 2023)). Differentially expressed genes (DEGs) were determined by using DESeq2. Moreover, we established criteria to screen significantly differential expression (False discovery rate (FDR < 0.01) and fold change (FC ≥ 2)). Enrichment analysis was carried out according to the hypergeometric test, with the pathway-based hypergeometric distribution being checked against the Kyoto Encyclopedia of Genes and Genomes (KEGG) and Gene Ontology (GO) term-based profiles.

### 4.3. Extraction, Separation, Identification and Quantification of Anthocyanins

In terms of the AB Sciex QTRAP 6500 LC-MS/MS platform, anthocyanin accumulations were detected by MetWare Biotechnology Company (Wuhan, China) (http://www.metware.cn/ (accessed on 10 July 2023)). First, about 0.05 g of powder was extracted with 0.5 mL of methanol/water/hydrochloric acid (500:500:1, *v*/*v*/*v*). Afterwards, the extract was vortexed for 300 s, subjected to ultrasound for 300 s and centrifuged at 12,000 r/60 s for 180 s at 4 °C; these steps were then repeated one more time. Afterwards, the supernatants were obtained and filtrated by utilizing a 0.22 μm membrane filter.

By utilizing an UPLC-APCI-MS/MS system (UPLC, ExionLC™ AD, https://sciex.com.cn/ (accessed on 12 July 2023) and MS, Applied Biosystems 6500 Triple Quadrupole, https://sciex.com.cn/ (accessed on 12 July 2023)), the sample extracts of anthocyanins were examined. Linear ion trap (LIT) and triple quadrupole (QQQ) scans were performed using a triple QTRAP, QTRAP^®^ 6500+ LC-MS/MS System equipped with an ESI Turbo Ion-Spray interface, which was run in the positive ion mode and managed by the Analyst 1.6.3 software (Sciex). By using scheduled multiple reaction monitoring (MRM), the content of anthocyanins was investigated. Data acquisitions were conducted through the Analyst 1.6.3 software (Sciex). Subsequently, we quantitatively evaluated metabolites by employing the Multiquant 3.0.3 software (Sciex). Mass spectrometric parameters, including declustering potentials (DPs) and collision energies (CEs) for individual MRM transitions, were generated via DP and CE optimization. Then, we detected the specific set of MRM transitions for each period. Notably, anthocyanin test results include both quantitative and semi-quantitative substances. Among them, the substances with standards were qualitatively quantified using standards, and the substances without standards were qualitatively quantified according to the mass spectrometry cleavage law, and the quantification was quantified via the calibration of analogs (anthocyanins were selected as delphinidin-3,5-O-diglucoside).

### 4.4. Extraction, Separation, Identification and Quantification of Carotenoids

Similarly, we extracted a total of 0.05 g of material by utilizing 0.5 mL of a solution that was formed by mixing N-Hexane, acetone and ethanol in equal proportions. Subsequently, we vortexed the extract for 20 min at about 20 °C. After being centrifuged at 12,000 r/min for 300 s at 4 °C, we picked up the liquid, and the above steps were repeated. Subsequently, the liquid was vaporized to dryness in a vacuum and rebuilt in a solution that was formed by mixing MeOH/MTBE in equal proportions, and the liquid was filtered.

In addition to the QTRAP^®^ 6500+ LC-MS/MS System equipped with an APCI Heated Nebulizer, the next steps and methods used were the same as those used for analyzing anthocyanins. Similarly, carotenoid test results also included both quantitative and semi-quantitative substances. Among them, the substances with standards were qualitatively quantified using standards, and the substances without standards were qualitatively quantified according to the mass spectrometry cleavage law, and the quantification was quantified by the calibration of analogs (carotenoids were selected as neoxanthin).

### 4.5. qRT-PCR Analysis

RNA was extracted and reverse-transcribed using a RNA pure plant kit (Tb Green^®^ Premix Ex Taq™ II (TAKARA, Beijing, China)) and Monad first-strand cDNA Synthesis Kit (SuperScript, Shanghai, China), respectively. Moreover, by utilizing the PRIMER-BLAST, we designed the primers (Table S1). In addition, we selected the gene (*MsG0380015289.01*) as the reference gene due to its high and steady expression levels in all samples based on the transcriptome data. Samples were placed in a real-time PCR machine and assays were set up on ABI Quant Studio 1 software. All results were obtained from three biological replicates. The primers are listed in Table S6.

## 5. Conclusions

In this work, we revealed the molecular mechanism of flower color formation in *M*. *sativa* ssp. *falcata* (purple flowers) and *M*. *sativa* ssp. *falcata* (yellow flowers) via a comparative analysis of the metabolome and transcriptome of three types of petals (purple, yellow and cream). The metabolome results demonstrated that anthocyanins (cyanidin, delphinidin, peonidin malvidin, pelargonidin and petunidin derivatives) are the critical pigments involved in the color formation of purple flowers, and the content of delphinidin-3,5-O-diglucoside and lutein was highest in purple and yellow flowers, respectively. Moreover, we found that twenty-five upregulated structural genes (one *C4H*, three *4CL*, twelve *CHS*, two *CHI*, one *F3H*, one *F3*′*H*, one *F3*′*5*′*H* and four *DFR*) may be crucial genes in the synthesis of anthocyanins in purple flowers. Simultaneously, we identified that carotenoids (α-carotene, β-carotene, (E/Z)-phytoene, α-cryptoxanthin, lutein and its derivatives, β-cryptoxanthin and its derivatives, zeaxanthin derivatives, violaxanthin and its derivatives, antheraxanthin and neoxanthin) play an essential role in the color formation of yellow flowers. Additionally, the transcriptome results revealed that nine structural genes (two *PSY*, one *ZDS*, two *CRTISO*, two *BCH*, one *ZEP* and one *ECH*) might participate in the synthesis of carotenoids in yellow flowers. Finally, we discovered that many differentially expressed TFs are associated with anthocyanin and carotenoid biosynthesis. This work provides insights into the molecular mechanism of flower color formation in *M*. *sativa* ssp. *falcata* and *M*. *sativa* ssp. *falcata*, and it has established a theoretical foundation for explaining the color diversity of alfalfa flowers.

## Figures and Tables

**Table 1:** DAMs of anthocyanins in the three comparison groups.

Group	Number of DAMs	Upregulated	Downregulated
P vs. C	42	33	9
Y vs. C	35	15	20
P vs. Y	51	38	13

**Table 2:** DAMs of carotenoids in the three comparison groups.

Group	Number of DAMs	Upregulated	Downregulated
P vs. C	20	11	9
Y vs. C	38	33	5
P vs. Y	39	32	7

## Data Availability

All data are open and available. The raw data are available in the NCBI database (BioProject ID PRJNA1045827) (https://www.ncbi.nlm.nih.gov/sra/URL (accessed on 28 December 2023)).

## Supplementary Materials

The following supporting information can be downloaded at: https://www.mdpi.com/article/10.3390/plants13050700/s1. Quantitative analysis of DAEs of anthocyanins among the three petals (P, Y and C groups) (Table S1); quantitative analysis of DAEs of anthocyanins between P and C (Table S2); quantitative analysis of DAEs of carotenoids among the three petals (P, Y and C groups) (Table S3); quantitative analysis of DAEs of carotenoids between Y and C (Table S4); data filtering and comparison of reference tables (Table S5); Primers used in qRT-PCR in this study (Table S6); the proportion of anthocyanins in the purple flowers (Figure S1); the proportion of carotenoids in the yellow flowers (Figure S2); hierarchical clustering of DEGs in the three samples of petals (Figure S3); volcano maps in (A) P vs. C, (B) Y vs. C, and (C) P vs. Y (Figure S4); KEGG enrichment map in (A) P vs. C and (B) Y vs. C (Figure S5); verification of ten DEGs by qRT-PCR (Figure S6).
