# Supplementary material for: Integrated Metabolomic and Transcriptomic Profiles Provide Insights into the Mechanisms of Anthocyanin and Carotenoid Biosynthesis in Petals of Medicago sativa ssp. sativa and Medicago sativa ssp. falcata
Source: Plants (Basel). 2024 Feb 29;13(5):700. doi: 10.3390/plants13050700 (PMC10934425; doi:10.3390/plants13050700)
Supplement: Supplementary file 1 [file plants-13-00700-s001.zip › Supplemental Figures.pdf]

Supplemental Figures

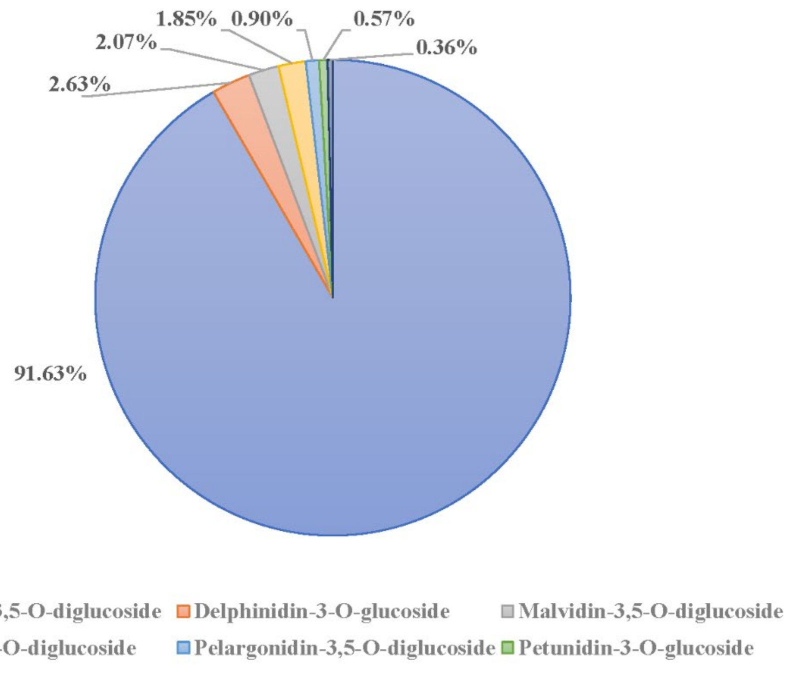

Supplemental Figure S1: The proportion of anthocyanins in the purple flowers.

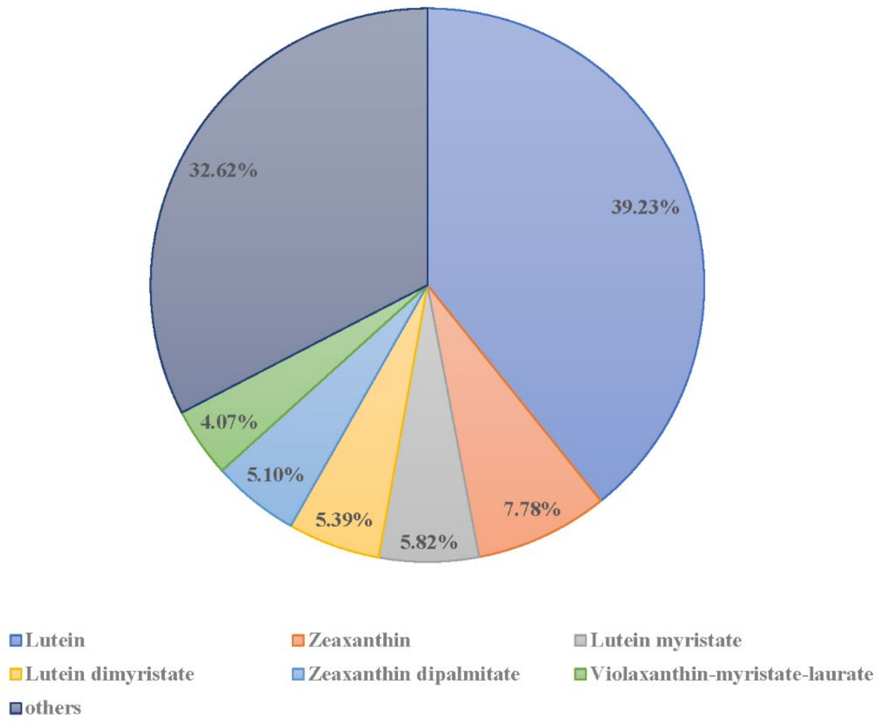

Supplemental Figure S2: The proportion of carotenoids in the yellow flowers.

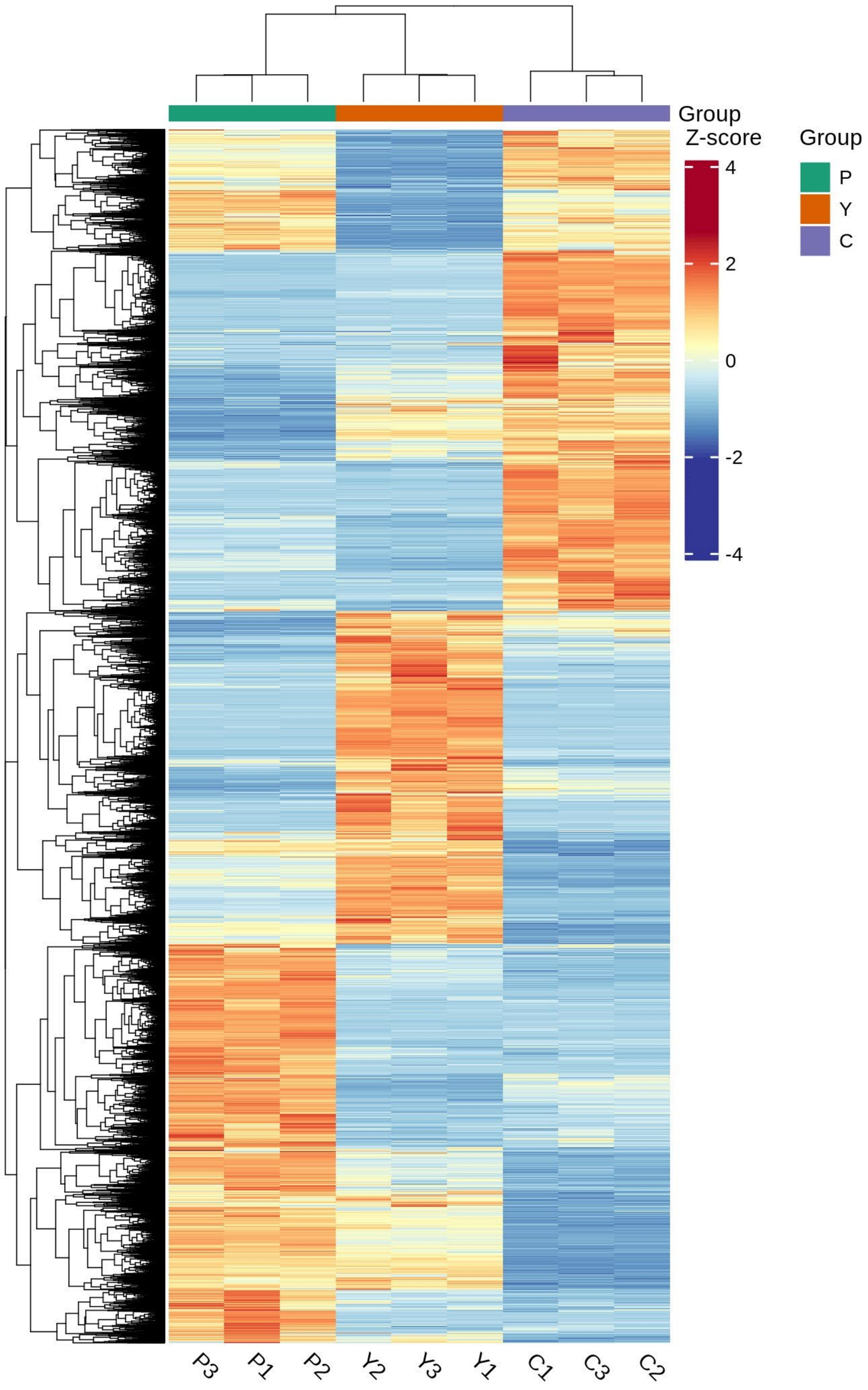

Supplemental Figure S3: Hierarchical clustering of DEGs in three petals. A low to high expression is suggested by color changes (blue to red).

A

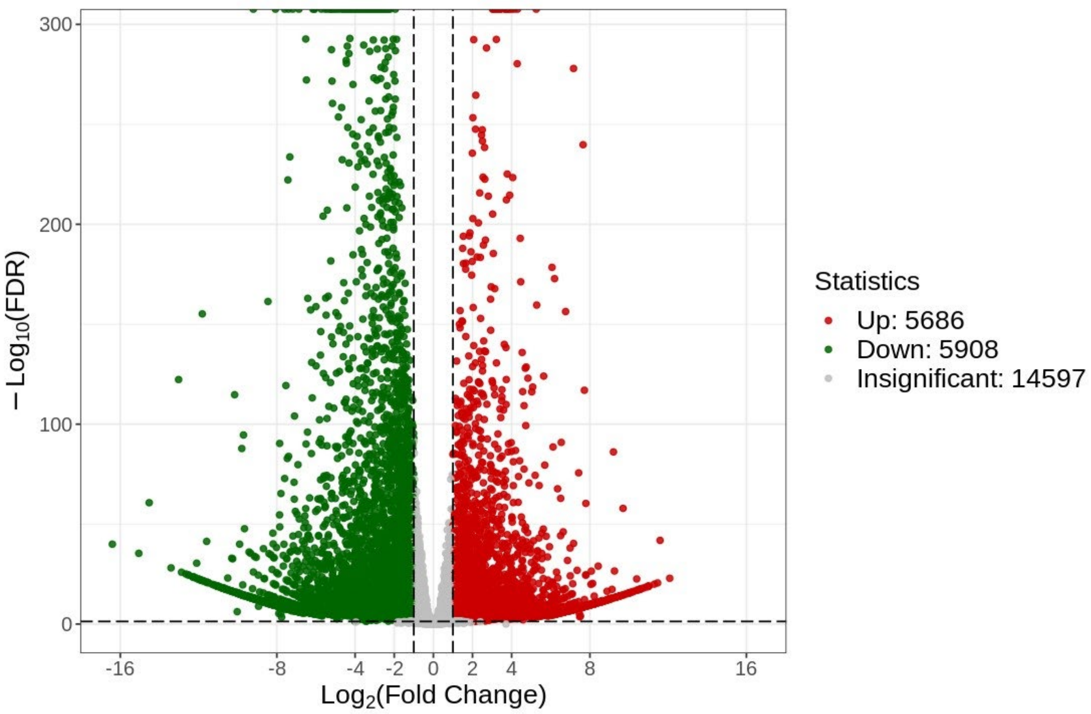

300
200
100
0
$-Log_{10}(FDR)$
-16
-8
-4
-2
0
2
4
8
16
Log_2(Fold Change)
Statistics
Up: 5686
Down: 5908
Insignificant: 14597

B

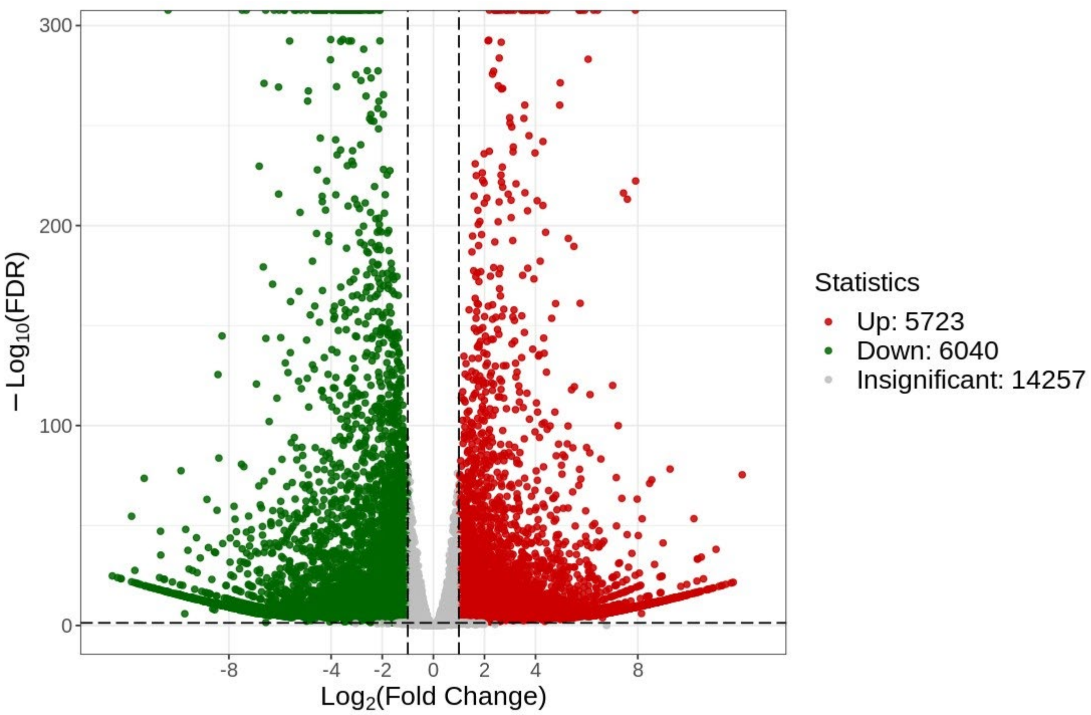

300
200
100
0
$-Log_{10}(FDR)$
-8
-4
-2
0
2
4
8
Log_2(Fold Change)
Statistics
Up: 5723
Down: 6040
Insignificant: 14257

C

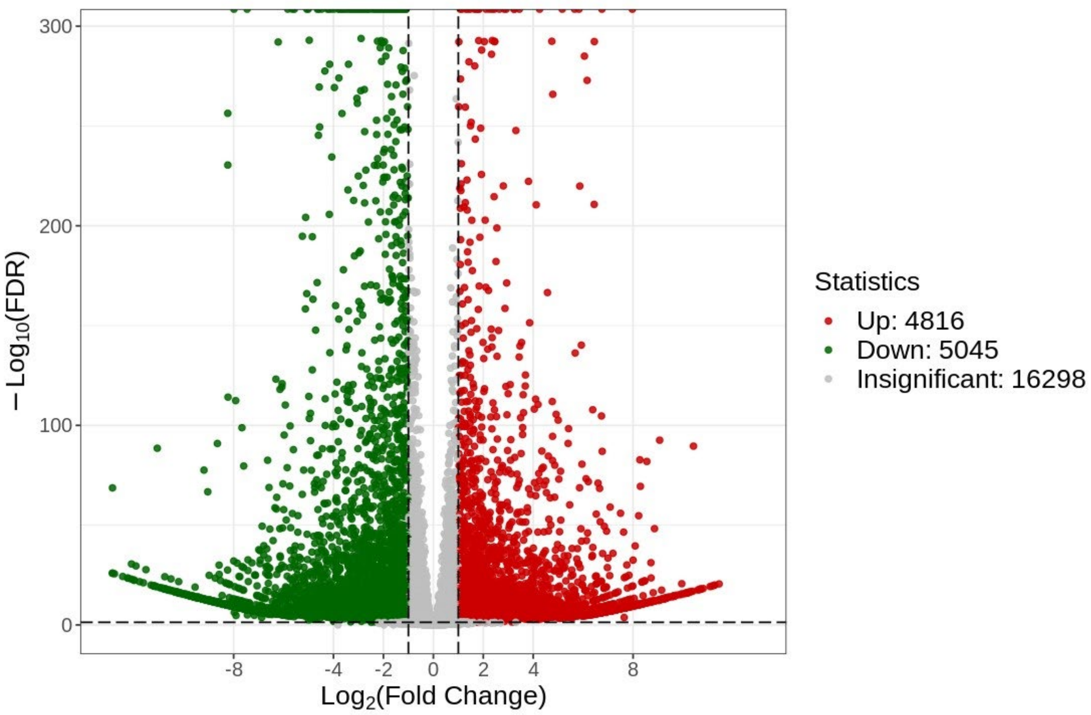

Supplemental Figure S4: Volcano maps in (A) P vs C, (B) Y vs C and (C) P vs Y

A

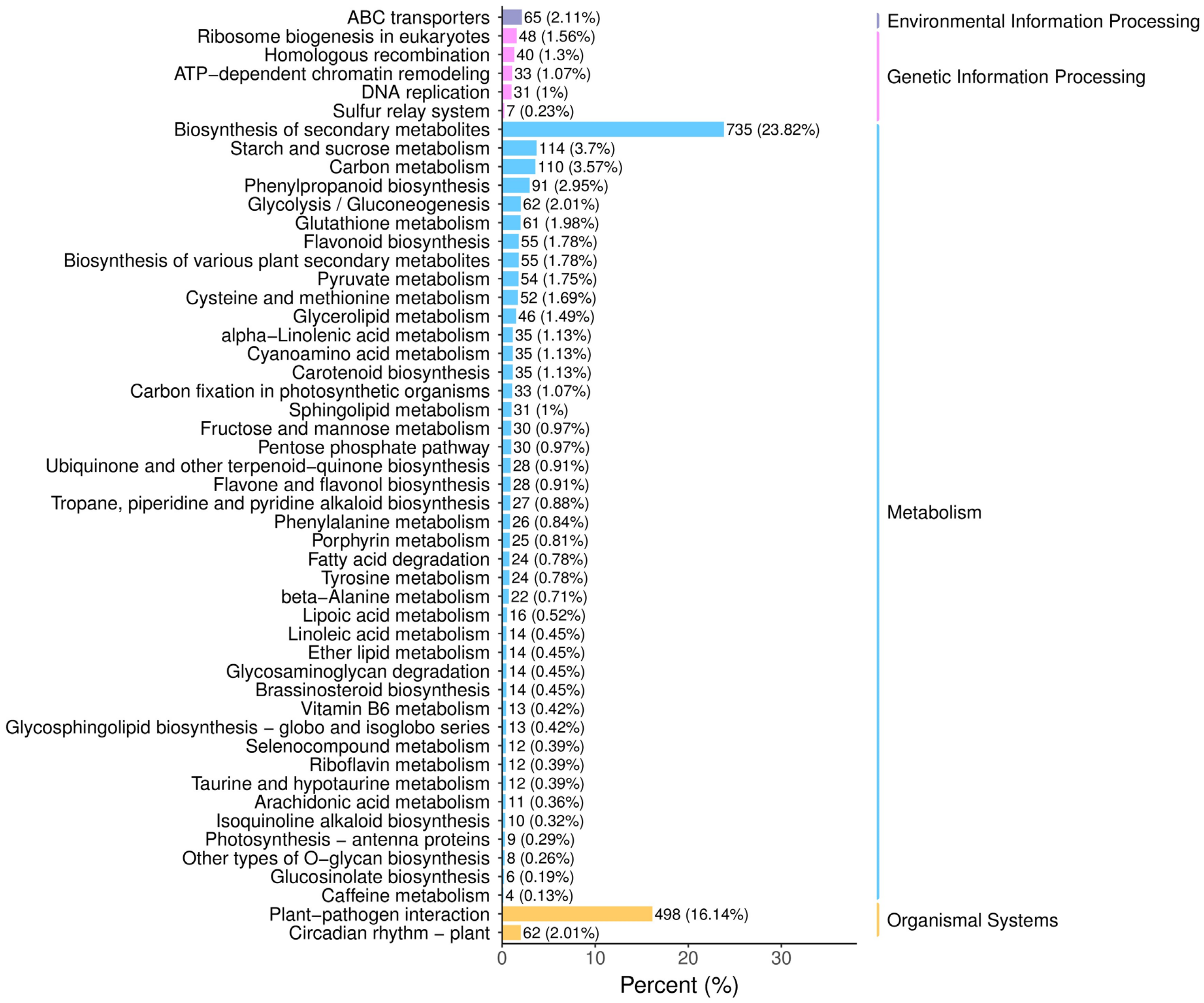

ABC transporters
65 (2.11%)
Ribosome biogenesis in eukaryotes
48 (1.56%)
Homologous recombination
40 (1.3%)
ATP−dependent chromatin remodeling
33 (1.07%)
DNA replication
31 (1%)
Sulfur relay system
7 (0.23%)
Biosynthesis of secondary metabolites
735 (23.82%)
Starch and sucrose metabolism
114 (3.7%)
Carbon metabolism
110 (3.57%)
Phenylpropanoid biosynthesis
91 (2.95%)
Glycolysis / Gluconeogenesis
62 (2.01%)
Glutathione metabolism
61 (1.98%)
Flavonoid biosynthesis
55 (1.78%)
Biosynthesis of various plant secondary metabolites
55 (1.78%)
Pyruvate metabolism
54 (1.75%)
Cysteine and methionine metabolism
52 (1.69%)
Glycerolipid metabolism
46 (1.49%)
alpha−Linolenic acid metabolism
35 (1.13%)
Cyanoamino acid metabolism
35 (1.13%)
Carotenoid biosynthesis
35 (1.13%)
Carbon fixation in photosynthetic organisms
33 (1.07%)
Sphingolipid metabolism
31 (1%)
Fructose and mannose metabolism
30 (0.97%)
Pentose phosphate pathway
30 (0.97%)
Ubiquinone and other terpenoid−quinone biosynthesis
28 (0.91%)
Flavone and flavonol biosynthesis
28 (0.91%)
Tropane, piperidine and pyridine alkaloid biosynthesis
27 (0.88%)
Phenylalanine metabolism
26 (0.84%)
Porphyrin metabolism
25 (0.81%)
Fatty acid degradation
24 (0.78%)
Tyrosine metabolism
24 (0.78%)
beta−Alanine metabolism
22 (0.71%)
Lipoic acid metabolism
16 (0.52%)
Linoleic acid metabolism
14 (0.45%)
Ether lipid metabolism
14 (0.45%)
Glycosaminoglycan degradation
14 (0.45%)
Brassinosteroid biosynthesis
14 (0.45%)
Vitamin B6 metabolism
13 (0.42%)
Glycosphingolipid biosynthesis − globo and isoglobo series
13 (0.42%)
Selenocompound metabolism
12 (0.39%)
Riboflavin metabolism
12 (0.39%)
Taurine and hypotaurine metabolism
12 (0.39%)
Arachidonic acid metabolism
11 (0.36%)
Isoquinoline alkaloid biosynthesis
10 (0.32%)
Photosynthesis − antenna proteins
9 (0.29%)
Other types of O−glycan biosynthesis
8 (0.26%)
Glucosinolate biosynthesis
6 (0.19%)
Caffeine metabolism
4 (0.13%)
Plant−pathogen interaction
498 (16.14%)
Circadian rhythm − plant
62 (2.01%)
0
10
20
30
Percent (%)
Environmental Information Processing
Genetic Information Processing
Metabolism
Organismal Systems

B

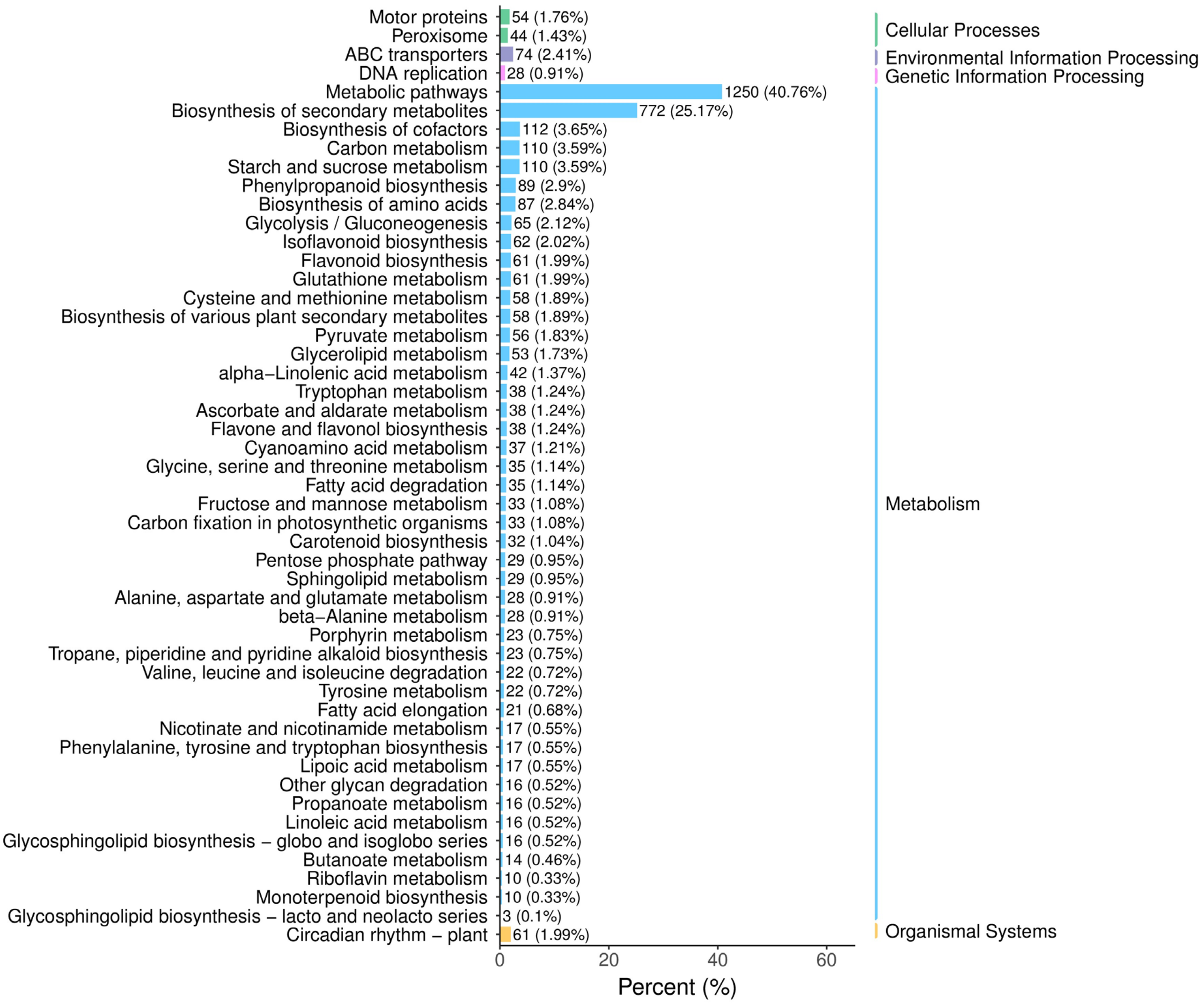

Supplemental Figure S5: KEGG enrichment map in (A) P vs C and (B) Y vs C.

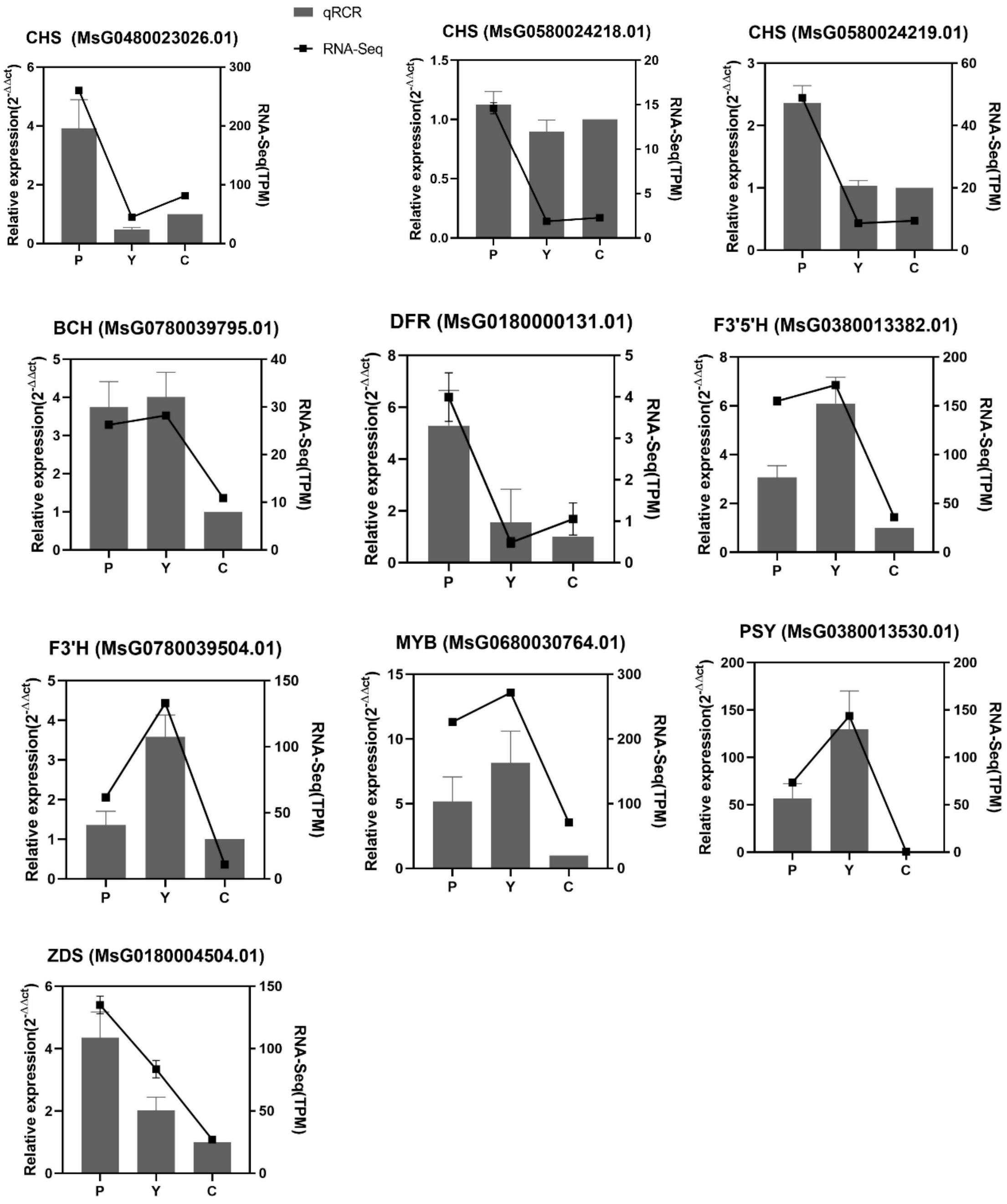

Supplemental Figure S6: Verification of ten DEGs by qRT-PCR. Data are the means of three replicates.
